# MicroRNA-200a Regulates Grb2 and Suppresses Differentiation of Mouse Embryonic Stem Cells into Endoderm and Mesoderm

**DOI:** 10.1371/journal.pone.0068990

**Published:** 2013-07-18

**Authors:** Yang Liu, Qidong Liu, Wenwen Jia, Jie Chen, Jianmin Wang, Dan Ye, Xudong Guo, Wen Chen, Guoping Li, Guiying Wang, Anmei Deng, Jiuhong Kang

**Affiliations:** 1 Department of Laboratory Medicine and Department of Hematology, Changhai Hospital, Second Military Medical University, Shanghai, People’s Republic of China; 2 Clinical and Translational Research Center of Shanghai First Maternity and Infant Health Hospital, Shanghai Key Laboratory of Signaling and Disease Research, School of Life Science and Technology, Tongji University, Shanghai, People’s Republic of China; Centro Cardiologico Monzino, Italy

## Abstract

The mechanisms by which microRNAs (miRNAs) affect cell fate decisions remain poorly understood. Herein, we report that miR-200a can suppress the differentiation of mouse embryonic stem (ES) cells into endoderm and mesoderm. Interestingly, miR-200a directly targets growth factor receptor-bound protein 2 (Grb2), which is a key adaptor in the Erk signaling pathway. Furthermore, high levels of miR-200a dramatically decrease Grb2 levels and suppress the appearance of mesoderm and endoderm lineages in embryoid body formation, as well as suppressing the activation of Erk. Finally, Grb2 supplementation significantly rescues the miR-200a-induced layer-formation bias and the Erk suppression. Collectively, our results demonstrate that miR-200a plays critical roles in ES cell lineage commitment by directly regulating Grb2 expression and Erk signaling.

## Introduction

Embryonic stem (ES) cells are derived from the inner cell mass (ICM) of blastocysts, one of the early stages of embryonic development. These cells retain the features of pluripotency and self-renewal while serving as the progenitors of all cell types [1–3]. The regulatory mechanism for the differentiation of ES cells into functional cells remains unclear. Therefore, an in-depth understanding of the molecular mechanisms of cell lineage differentiation will facilitate clinical applications of stem cell therapy [4]. MicroRNAs (miRNAs), which are 21-23 nucleotide non-coding RNAs [5,6], have been identified as a class of gene regulators that act during the individual development and differentiation of specific cell types [7,8]. In the canonical pathway of miRNA biogenesis, the primary miRNAs processed into Drosha-DiGeorge syndrome critical region gene 8 (DGCR8) complexes to produce pre-miRNAs [9–13]. The pre-miRNAs are then transported into the cytoplasm by Exportin-5 [14–16] and are further processed into mature miRNAs by Dicer [17–20]. miRNAs are incorporated into the RNA-induced silencing complex (RISC), which then localizes to the 3’ untranslated region (UTR) of the target mRNA [21,22], leading to gene silencing [23–26] or degradation [27] at a post transcriptional level. miRNAs are a determinant of ES cell characteristics in early developmental processes [28].

Previous studies show that miR-200 family members are emerging as important regulators of cell proliferation, differentiation and metastasis [29–31]. The miR-200 family consists of five members (miR-200a, -200b, -200c, -141 and -429) that are expressed as two separate polycistronic pri-miRNA transcripts. The sequences encoding miR-200b/200a/429 exist as a cluster on mouse chromosomes 4, and those encoding miR-200c/141 exist as a cluster on chromosome 6 [32]. In previous studies, miR-200 family members were shown to promote the mesenchymal-epithelial transition (MET) and to activate the differentiation of pancreatic, colorectal and breast cancer cells into epithelial cells [33–35]. miR-200 family members directly target Zeb1/Zeb2 and enhance E-cadherin expression, resulting in the suppression of murine mammary tumor cell migration [33,36]. In contrast, Zeb1 suppresses the expression of miR-200 family members, forming a regulatory feedback loop [37]. A recent study demonstrated that miR-200a overexpression prevents the transformation of normal mammary cells and decreases cell migration by targeting the class III histone deacetylase silent information regulator 1 (Sirt1) [38]. miR-200a also targets p38alpha and regulates the oxidative stress response, affecting tumorigenesis and chemosensitivity [39]. miR-200a overexpression decreases Smad-3 activity and the matrix protein, including Collagen I, Collagen IV and Fibronectin, blocks the TGF-beta dependent epithelial-mesenchymal transition (EMT) process, and rescues early and advanced kidney disease in mouse models [40]. However, the function of miR-200a in the initiation of vertebrate embryo development has not been reported (A list of miR-200 family members targets in stem cells and development is shown in Table 1).

**Table 1:** miR-200 family menbers in stem cells and development.

Target	Member	Cell type	References
Zeb1/Zeb2	miR-200a, miR-200b, miR-200c, miR-429, miR-141	Induced pluripotent stem cells, breast cancer stem cells, neuroectodermal precursors	Wang et al. 2013 [70], Radisky 2011 [71], Du et al. 2013 [72]
Bmi1	miR-200c	embryonal carcinoma cells	Shimono et al. 2009 [73]
Sox2	miR-200c, miR-141	neural stem/progenitor cells	Peng et al. 2012 [29]
E2F3	miR-200c, miR-141	neural stem/progenitor cells	Peng et al. 2012 [29]
Suz12	miR-200b	Breast cancer stem cells	Iliopoulos et al. 2010 [74]
Flk1	miR-200c	embryonic stem cells	Gill et al. 2012 [75]
Ets1	miR-200c	embryonic stem cells	Gill et al. 2012 [75]
Srf	miR-200b	oligodendrocyte progenitor cells	Buller et al. 2012 [76]

Growth factor receptor-bound protein 2 (Grb2) is a key adapter protein in intracellular signal transduction pathways, in which it links activated cell surface receptors to downstream targets by binding to specific phosphotyrosine-containing and proline-rich sequence motifs [41,42]. Deletion of the Grb2 gene leads to preimplantation embryonic lethality in vivo [43,44]. Signaling via Grb2 is essential to the segregation of epiblast and primitive endoderm progenitors [43]. Those findings suggest that Grb2 might support differentiation. However, the roles of Grb2 and miRNA-induced intracellular signaling cascades in lineage commitment are not well understood.

In this paper, we report that miR-200a was highly expressed in ES cells and gradually decreased in expression during embryoid body (EB) formation and that miR-200a suppressed endoderm and mesoderm lineage commitment. We further identified Grb2 as a direct regulatory target of miR-200a. In EB, knockdown of Grb2 with a specific shRNA had an identical effect to treatment with miR-200a. Finally, a rescue assay showed that exogenous Grb2 could reverse the miR-200a-induced endoderm and mesoderm suppression. Similarly, the extracellular signal-regulated kinase (Erk) signaling, when activated with assistance from Grb2, also rescued miR-200a-induced effects. Taken together, these results suggest that miR-200a might control cell fate decisions affecting the early endoderm and mesoderm layers in a manner that is partly dependent on Erk signaling, by regulating Grb2 expression levels.

## Materials and Methods

### Cell culture

The mouse ES cell line ES-E14TG2a, purchased from the American Type Culture Collection (ATCC CRL-1821), was maintained on gelatin-coated plates in high glucose-Dulbecco’s Modified Eagle Medium (DMEM; Gibco) that was supplemented with 15% (vol/vol) ES qualified-fetal bovine serum (FBS; Gibco), 2 mM L-glutamine (Hyclone), 1x nonessential amino acids (Hyclone), 50 mM beta-mercaptoethanol (Gibco) and 500-1000 U/ml of leukocyte inhibitory factor (LIF; generated in house). ES cells were trypsinized and split every 2 days, and the culture medium was changed daily. For the formation of EB, ES cells were plated in 3.5 cm dishes at a density of 5 × 10^4^ cells in 2 ml medium without LIF. HEK293T cells were cultured in DMEM (Gibco) that was supplemented with 100 U/ml of penicillin/streptomycin (Invitrogen) and 10% FBS (Gibco).

### Transfection of miRNA mimics and inhibitors

miR-200a mimics and inhibitors (including the Negative control) were purchased from Ribo. ES cells were seeded into 6-well plates at a density of 5 × 10^4^ cells per well. For miRNA overexpression or knockdown experiments, miRNA mimics or inhibitors and the scrambled negative control were gently mixed with Opti-MEM (Gibco) and X-tremeGENE 9 Transfection Reagents (Roche) according to the manufacturer’s instructions. At 12-72 hours post-transfection, the cells were harvested for Western blotting or quantitative real-time PCR analysis.

### Plasmids

The miR-200a sequence was obtained from the mirbase website and cloned into the AgeI and EcoRI sites of the pLKO.1 vector. To construct a miR-200a sponge vector, the following oligoribonucleotides for the miR-200a sponge were designed and synthesized: miR-200a sponges forward oligo, CCGGTACATCGTTACTCTCAGTGTTACCGACATCGTTACTCTCAGTGTTAGCGACATCGTTACTCTCAGTGTTAG; reverse oligo, AATTCTAACACTGAGAGTAACGATGTCGCTAACACTGAGAGTAACGATGTCGGTAACACTGAGAGTAACGATGTA. After an annealing step at 95 °C for 4 min, the sponge oligoribonucleotides were inserted into the pFUW plasmid between the BamHI and AgeI sites. For luciferase reporter assays, a pGL3-Grb2-3’UTR-WT vector was constructed. A 3’ UTR segment of wild-type Grb2 mRNA, which contained the putative target sites of miR-200a, was amplified and cloned into the SacI and XbaI sites downstream of the luciferase reporter gene in pGL3. A pGL3-Grb2-3’UTR-Mutant vector, which carried a mutation in the complementary site for the seed region of miR-200a, was generated from the pGL3-Grb2-3’UTR-WT vector by mutation PCR. The Grb2 expression vector was created by cloning the Grb2 coding sequence into the BamHI and AgeI sites of pFUW.

### Protein analysis

Cells were lysed in RIPA buffer that contained a protease inhibitor cocktail. Estimation of lysate protein concentrations was performed with a BCA Protein Assay Kit (Pierce). Approximately 100 µg of lysate was resolved on a 12% SDS/PAGE gel and transferred to NC membranes (Bio-Rad). The membranes were blocked with blocking solution (3% BSA in TBST) and incubated with primary antibodies. The following primary antibodies were used: mouse anti-Oct4 (1:1000; Santa Cruz Biotechnology), rabbit anti-Sox2 (1:1000; Millipore), rabbit anti-Nanog (1:1000; Abcam), rabbit anti-Grb2 (1:1000; Bioworld), rabbit anti-Erk (1:1000; Bioworld), rabbit anti-p-Erk (1:1000; Cell signaling) and rabbit anti-GAPDH (1:1000; Miaotong). The blots were subsequently incubated with either HRP-conjugated anti-rabbit IgG or HRP-conjugated anti-mouse IgG (1:3000; Cell signaling). Labeled proteins were detected with the eECL Western Blot Kit (Cwbio).

### Quantitative real-time PCR

Total RNA was extracted from ES cells and EBs with TRIzol reagent (Takara) according to the manufacturer’s instructions. cDNA synthesis was performed from 250 ng of total RNA in a single step (37 °C for 15 min) with the Takara reverse transcription kit. Quantitative real-time PCR was performed with iTaq reagent (Bio-Rad) and a STRATAGENE Mx3005p Real-Time PCR Cycler. The following PCR cycle conditions were used: initial denaturation for 30 sec at 95 °C, followed by 40 cycles of 5 sec at 95 °C and 30 sec at 60 °C. Mature miRNA primers, including stem-loop RT and special miRNA primers, were purchased from Ribo. Real-time PCR primers are shown in Table S1.

### Dual luciferase reporter assay

At 12 hours prior to the transfections, HEK293T cells were plated at a density of 5 × 10^4^ cells per well in 24-well plates. Cells were transfected via Lipofectamine 2000 Transfection Reagent (Invitrogen) and Opti-MEM (Gibco) with 50 nM miRNA mimics, 400 ng of the luciferase vector (pGL3 constructs) and 20 ng of the Renilla vector. At 24 hours post-transfection, the cells were harvested, and the luciferase activity was measured with a dual-luciferase reporter assay (Promega) according to the manufacturer’s instructions.

### Alkaline phosphatase staining

ES cells were cultured at a clonal density (3000 cells/cm^3^). Three days later, ES cells were fixed in 4% paraformaldehyde for 3 min at room temperature and then rinsed in PBS solution for 5 min. ES cells were stained with a staining solution of with 0.4% N,N-dimethylformamide (Sigma) and 0.06% Red Violet LB salt (Sigma). The result was obtained after 30 min of incubation at room temperature.

### Immunofluorescence staining

Cells were fixed with 4% paraformaldehyde for 20 min, washed three times with PBS, permeabilized with 0.1% Triton X-100 (Amresco) for 8 min, and blocked by incubation with 10% FBS (Gibco) in PBS for one hour to prevent nonspecific binding. The cells were incubated overnight at 4 ^°^C with the primary antibodies anti-alpha-fetoprotein (Afp; Sigma), anti-alpha-smooth muscle actin (alpha-Sma; Sigma), anti-Nestin (Millipore) and anti-Tuj1 (Millipore), which were diluted 1:1000 in PBS with 10% FBS (Gibco). Subsequently, cells were incubated for one hour at room temperature with the secondary Alexa Fluor 546-conjugated antibody (Invitrogen). The cells were counterstained with Hoechst 33342 to visualize the cell nuclei. Images were captured with a Nikon Eclipse Ti–S fluorescence microscope.

## Results

### Overexpression of miR-200a in ES cells suppressed differentiation into endoderm and mesoderm

**Figure 1:**
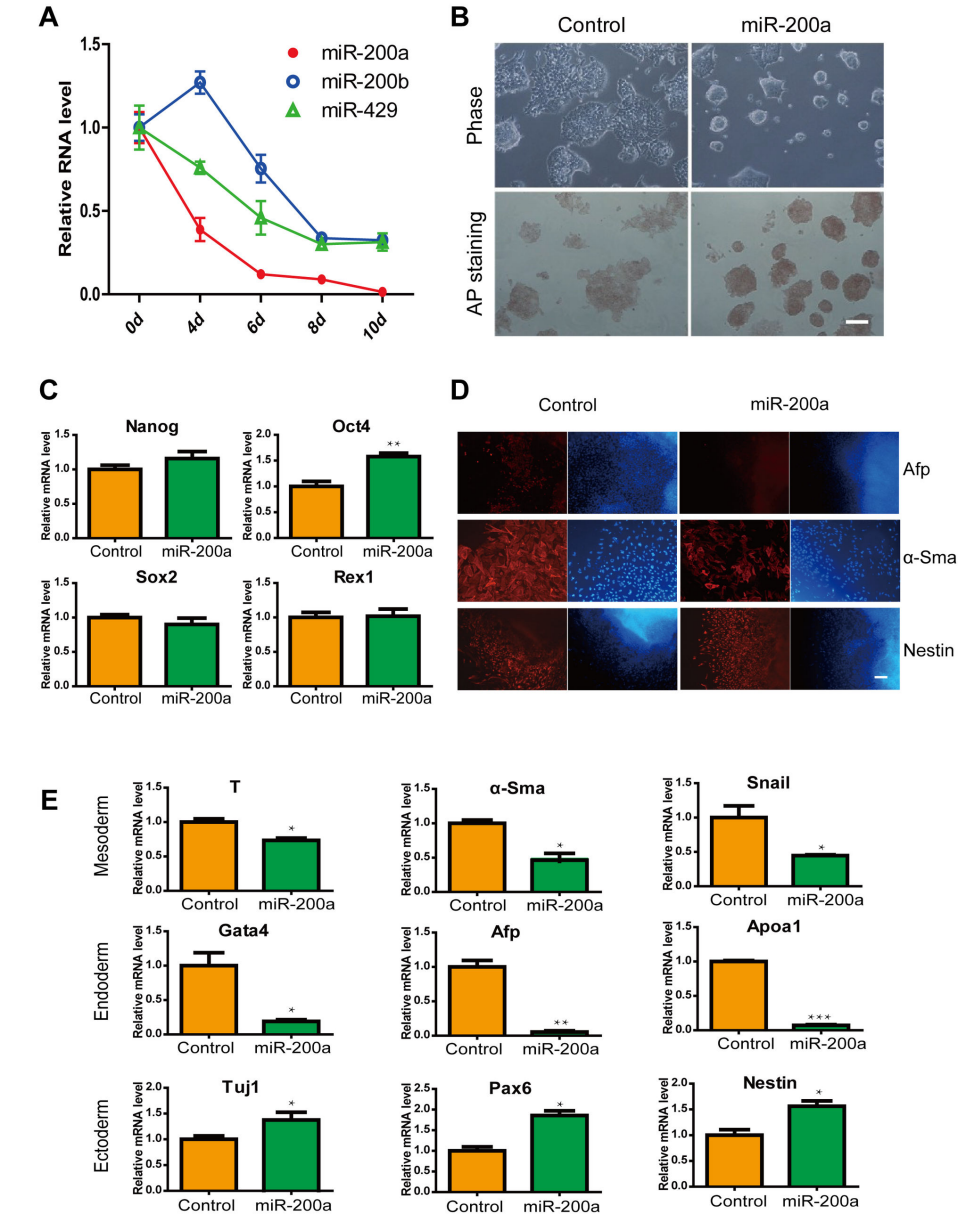
Effects of miR-200a in ES cells and ES cell differentiation. (A) The expression of miR-200a, miR-200b and miR-429 in ES cell diferentiation. (B) Brightfield images and alkaline phosphatase staining of ES cells without LIF at 72 hours post-transfection with miRNA-200a. The scale bar represents 100 µm. (C) Relative levels of Oct4, Nanog, Sox2 and Rex1 mRNA in control or miR-200a-transfected ES cells. (D) Representative immunofluorescence images of control and miR-200a overexpression after 10 days of EB formation. Red, layer markers; blue, nuclei. The scale bar represents 100 µm. (E) Expression levels of genes associated with the differentiated state in EBs in response to miR-200a expression. All data are shown as the means ± SD. Statistical significance was assessed by the two-tailed Student’s t test. ***, p < 0.001; **, p < 0.01; *, p < 0.05.

To determine whether miR-200 family is responsive to ES cell differentiation, we analyzed miR-200 mumbers (miR-200a, miR-200b and miR-429) expression during ES cell spontaneous differentiation by quantitative real-time PCR. We found that miR-200a expression was markedly decreased during EB formation (Figure 1A). To examine the role of miR-200a in the early differentiation processes of ES cells, we overexpressed miR-200a and performed differentiation experiments by withdrawing LIF 3 days. We observed that miR-200a promoted retention of the ES cell-like morphology and had high alkaline phosphatase activity at the epiblast-like stem cell stage. In contrast, control ES cell colonies tended to become flat and alkaline phosphatase activity was lower (Figure 1B). Additionally, we found that miR-200a increased expression of the self-renewal-associated gene Oct4 and maintained the expression of Nanog (Figure 1C). These results were identical to those in previous studies [45]. Interestingly, it has been reported that Oct4 expression is limited in the ICM and is finally downregulated in the primitive endoderm [46]. We supposed that miR-200a would be involved in the formation of the endoderm and other layers. To study the effects of miR-200a on spontaneous differentiation, we analyzed marker genes for the three layers. We found that miR-200a decelerated the process of ES cell differentiation, especially into the endoderm and mesoderm layers, as determined by the expression of marker genes. Immunostaining demonstrated that miR-200a-ES cell differentiation expressed low levels of alpha-Sma and Afp proteins (Figure 1D). In mRNA level, miR-200a reduced the expression of Brachyury (T), alpha-Sma, Snail, Afp, Gata4 and Apoa1. These ES cells failed to become mesoderm and endoderm cell. In contrast, expression levels of the ectodermal specific markers Tuj1, Pax6 and Nestin were increased in miR-200a treatment comparing to control (Figure 1E). Our findings demonstrated that miR-200a maintained ES cell pluripotency and suppressed differentiation capacity of endoderm and mesoderm.

### Grb2 as a novel and important target gene for miR-200a

**Figure 2:**
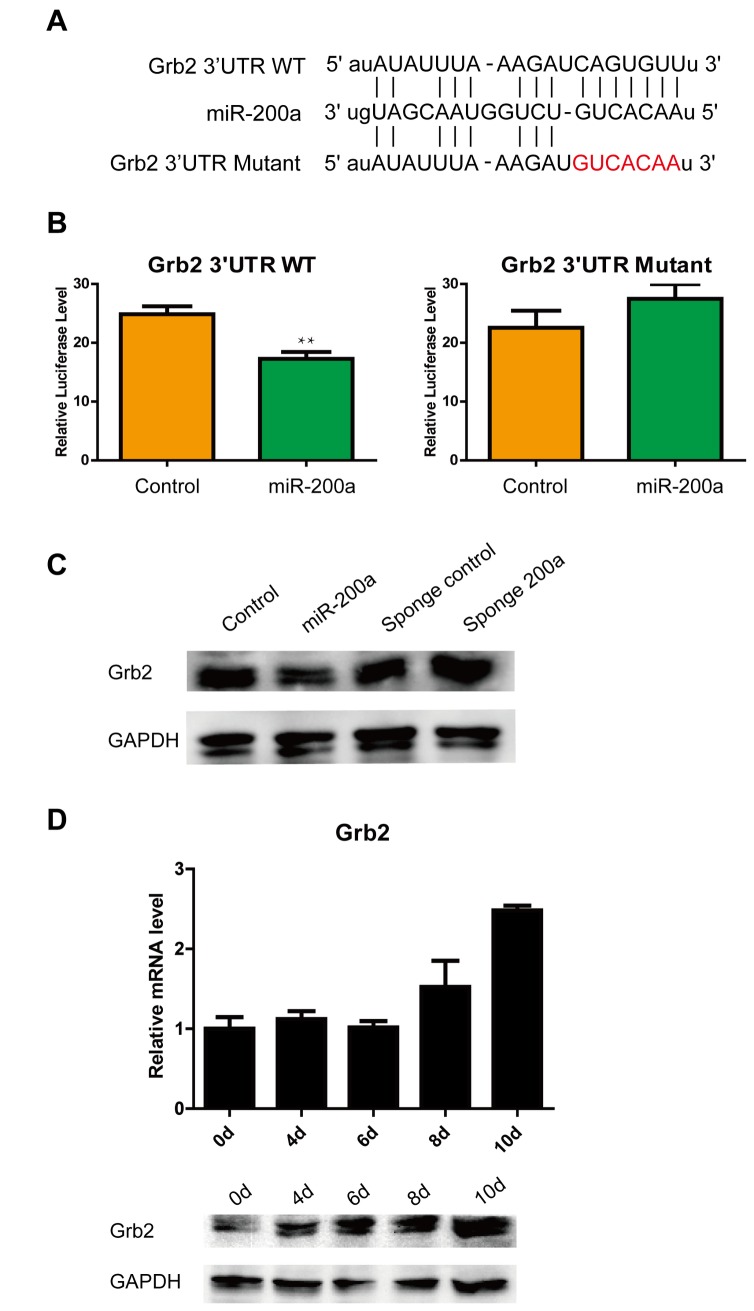
miR-200a targets Grb2 at the protein level. (A) Outline of the interaction of miR-200a with the Grb2 3’ UTR. (B) Repression of luciferase activity validates the interaction between miR-200a and the specific predicted sites in the Grb2 3’ UTR. (C) Overexpression of miR-200a downregulates Grb2 expression, and sponge 200a rescues Grb2 expression in ES cells. (D) The expression of Grb2 in ES cell diferentiation. All data are shown as the means ± SD. Statistical significance was assessed by the two-tailed Student’s t test. **, p < 0.01.

It was not clear whether miR-200a exerted its effects by negatively regulating multiple genes that are involved in ES cell identity. Grb2 had been suggested to be a putative target for miR-200a according to the miRDB, miRanda, TargetScan, PicTar and miRWalk algorithms, as well as a recent study [47]. The predicted interaction between the Grb2 3’ UTR and miR-200a is illustrated in Figure 2A. To confirm the post-translational repression of miR-200a, Grb2 3’ UTR reporter luciferase assays were performed. The delivery of miR-200a mimics significantly suppressed Grb2 3’ UTR reporter luciferase activity more than 25% over the empty vector, and a mutation in the miR-200a binding site blocked this suppression (Figure 2B). Additionally, we performed transient transfections of the miR-200a vector and sponge 200a vector into ES cells at a ratio of 3000 ng vectors per 5 × 10^4^ cells to investigate the regulation of Grb2. At 48 h post-transfection, Western blotting analysis showed that miR-200a reduced the level of endogenous Grb2 protein, whereas the Grb2 protein level was rescued from endogenous miR-200a by the sponge 200a treatment of ES cells (Figure 2C Figure S1A). Furthermore, Grb2 expression was upregulated during EB formation, unlike miR-200a expression (Figure 2D). These findings indicated that miR-200a specifically bound the predicted translation repressor site on Grb2 and repressed the expression of Grb2 in ES cells.

### Knockdown of Grb2 overlaps phenotypically with the enforced expression of miR-200a

**Figure 3:**
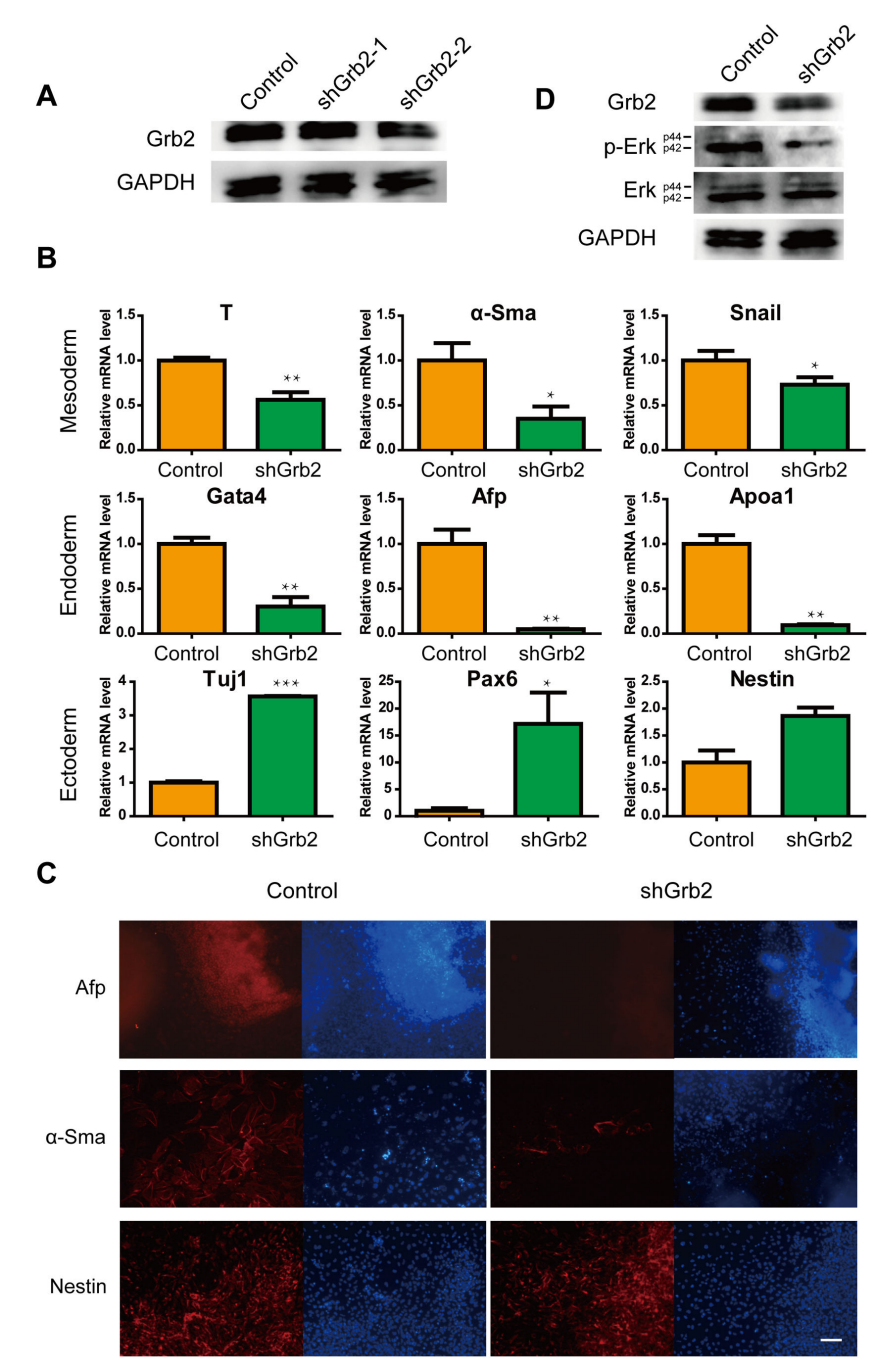
Knockdown of Grb2 during EB formation. (A) Western blotting analysis demonstrates that shGrb2 knocks down Grb2 protein levels in ES cells. (B) Relative levels of layer markers are varied in shGrb2-treated EBs. (C) The in situ expression of layer markers in day 10-differentiated EBs. Red, layer markers; blue, nuclear DNA staining. The scale bar represents 100 µm. (D) Erk activity decreases in response to Grb2 knockdown. All data are expressed as the means ± SD. Statistical significance was assessed by the two-tailed Student’s t test. ***, p < 0.001; **, p < 0.01; *, p < 0.05.

To determine whether the knockdown of Grb2 affected layer formation, we established two candidate Grb2 shRNA vectors, shGrb2-1 and shGrb2-2. shGrb2-1 did not significantly affect Grb2 expression. However, the results demonstrated that shGrb2-2 effectively reduced Grb2 protein expression (Figure 3A Figure S1B), and thus shGrb2-2 was used in subsequent studies.

To evaluate the effects of Grb2 during differentiation, we collected RNA at day 10 and performed quantitative real-time PCR for lineage-specific marker analysis. Gene expression analysis showed that when Grb2 expression was knocked down, changes in the expression of genes associated with differentiation were observed. Expression levels of genes related to differentiated states were significantly changed. The expression levels of genes associated with endoderm formation, such as Gata4, Afp and Apoa1, were downregulated. The induction of genes involved in mesoderm specification, such as T, alpha-Sma and Snail, was decreased in Grb2-deficient EBs, compared to those with scrambled controls. However, expression levels of the neuronal cell markers, Tuj1 and Pax6 were upregulated in response to Grb2 knockdown (Figure 3B).

As expected, the immunostaining of proteins associated with differentiation genes showed the same results. Grb2 shRNA and control EBs were plated at spontaneous differentiation day 10. We performed immunofluorescent staining and observed that the Afp and alpha-Sma protein levels were significantly decreased in the Grb2 shRNA EBs (Figure 3C). Grb2 is known to be involved in the Erk signaling pathway, and thus we measured the state of this signaling pathway to survey the role of Grb2 in ES cell differentiation. Similar to previous findings in tumors [48] and Grb2-null embryos [49], Grb2 knockdown led to reduced levels of phosphorylated Erk in spontaneous differentiated EBs (Figure 3D Figure S1C). Taken together, these data indicate that the loss of Grb2 suppressed differentiation toward the endoderm and mesoderm lineages under spontaneous differentiation conditions.

### Neutralization of Grb2 rescues aberrant miR-200a-induced endoderm and mesoderm repression

**Figure 4:**
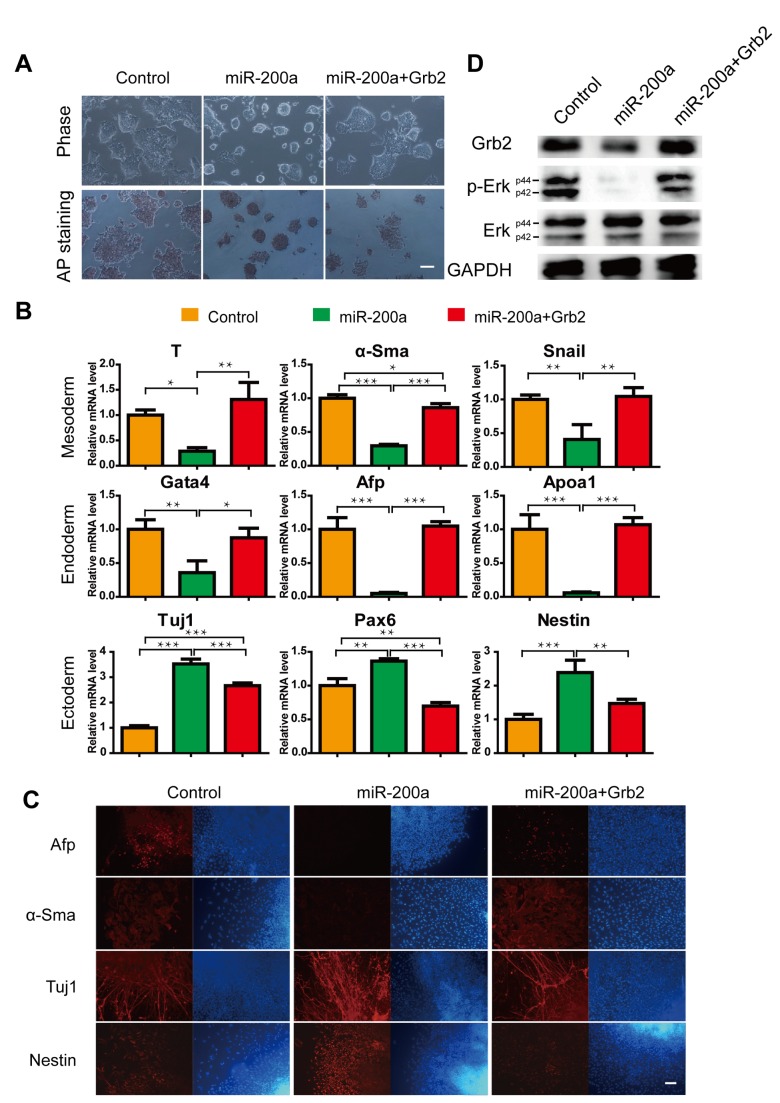
Grb2 can rescue cells from the effects of miR-200a. (A) Brightfield images and AP staining of wild-type ES cells, miR-200a-expressing ES cells and exogenous Grb2-expressing ES cells plus expressing miR-200a. The scale bar represents 100 µm. (B) Expression levels of genes associated with the differentiated state in EBs. (C) Immunofluorescence shows that Grb2 reversed the miR-200a-induced failures in endoderm and mesoderm differentiation. The scale bar represents 100 µm. (D) Erk activity can be rescued by Grb2 in miR-200a-expressing ES cells. All data are expressed as the means ± SD. Statistical significance was assessed by one-way ANOVA, followed by Tukey’s post-test. ***, p < 0.001; **, p < 0.01; *, p < 0.05.

To investigate whether the Grb2-miR-200a interaction is needed for the spontaneous differentiation of ES cells in vitro, ES cells were infected with a lentiviral vector that overexpressed Grb2 without the 3’ UTR. The cells were subsequently transfected with a miR-200a vector, followed by the immediate induction of spontaneous differentiation. We plated the ES cells at a density of 10^3^ cells/cm^2^ in the absence of LIF for 3 days. Under these conditions, in which miR-200a expression was rapidly induced, the Grb2 protein expression levels significantly decreased. An analysis of colony formation showed that ES cells that overexpressed miR-200a appeared to retain the classic compact morphology and well-defined borders of undifferentiated ES cell colonies. However, the colonies that overexpressed both Grb2 and miR-200a were flat and displayed abundant cytoplasmic prolongations when compared to the empty-vector control ES cells (Figure 4A). Next, we performed alkaline phosphatase staining to confirm this result. Only the miR-200a-overexpressing ES cell colonies showed strong staining; the majority of the control and Grb2/miR-200a-overexpressing ES cell colonies showed faint or no staining under the same conditions.

Interestingly, during EB formation, lower levels of the primitive endoderm markers Afp, Gata4, Apoa1 and the mesoderm markers T, alpha-Sma and Snail were observed in response to miR-200a overexpression, as well as opposite effects on the ectoderm markers Tuj1, Pax6 and Nestin. By contrast, the overexpression of Grb2 significantly rescued the expression of those genes (Figure 4B).

Furthermore, we performed immunofluorescent staining to examine the effects of the Grb2 expression constructs on ES differentiation. Consistently, miR-200a-overexpressing ES cells displayed much lower Afp, Gata4 and alpha-Sma expression levels and higher Tuj1 and Nestin expression levels. In contrast, Grb2-overexpressing ES cells had similar expression levels of the above-mentioned genes to the control cells (Figure 4C). This finding suggested that the overexpression of Grb2 partially reversed the changes in morphology and losses of endoderm and mesoderm formation that were induced by miR-200a.

To confirm the function of Grb2, we analyzed the activation of the downstream signaling protein Erk. In miR-200a-treated cells, both the Grb2 protein and the Erk phosphorylation levels were low. When we restored Grb2 expression, Erk phosphorylation was restored (Figure 4D Figure S1D). This finding suggested that Erk activation is controlled by miR-200a via the repression of Grb2 and that the main defect in miR-200a-induced endoderm and mesoderm formation was due to decreased Erk activation in response to reduced Grb2 levels.

## Discussion

During early embryogenesis, specific miRNAs have been shown to be essential for the maintenance of bias-in-fate decisions [50]. miR-206 promotes mesendoderm formation by targeting Prickle1a, which subsequently regulates Jnk2 phosphorylation, thereby indicating the potential function of miR-206 in embryonic axis formation [51]. miR-1 and miR-133 are essential to cardiac and skeletal muscle development [28]. During the differentiation of ectoderm, Let-7, miR-9 and miR-124a are specifically required for neuron production [52,53]. miR-124 targets Ptb and switches the balance between the expression of Ptb and nPtb to promote neuronal differentiation [54]. In our study, we found that miR-200a acted as an inhibitor of endoderm and mesoderm formation by repressing the expression of genes involved in mesoderm and endoderm formation. In contrast, ectoderm genes were enhanced in response to miR-200a. To further address the mechanisms underlying the effects of miR-200a, we predicted that Grb2 was a target of miR-200a repression and confirmed this by using a partial-length Grb2 3’ UTR reporter. The addition of miR-200a or Sponge 200a to ES cells further validated the potent and specific miR-200a-Grb2 connection at the protein level. To extend these findings, we investigated the effects of Grb2 knockdown and miR-200a overexpression, and we found that these induced similar characteristics during EB differentiation. Our data suggested that the effects of miR-200a might depend on the repression of Grb2.

Grb2 is an adapter protein that participates in the fibroblast growth factor (FGF) receptor signaling pathway and thus is involved in multiple aspects of cellular function. FGF, which controls the responsiveness of differentiation regulators, is of particular importance to mesoderm and endoderm formation [55]. Gene knockout experiments show that FGFR1 [56], FGFR2 [57] and FGF4 [58] mutant embryos fail to develop a primitive endoderm layer and die in blastocyst outgrowths or in vivo. Similarly, expression of negative FGF receptors does not contribute to the endoderm in chimeras due to a failure of the primitive streak [59]. On the other hand, FGF signaling in early embryogenesis initiates the activation of transcription factors that function in the differentiation of the endoderm and mesoderm layers. Our findings showed that, in response to Grb2 knockdown or miR-200a overexpression, Gata4 and Afp expression was decreased. Gata4 expression is upregulated by exogenous FGF1 in response to cardiac genes in differentiating embryonic carcinoma cell cultures [43], and Afp expression is dependent on FGFs that are produced by the cardiac mesoderm in embryonic endoderm cells [49]. Expression of the mesoderm marker T was also reduced; T is positively regulated by FGFR1 and controls mesodermal morphogenesis [60]. Similarly, decreases in Alpha-Sma expression were observed; in *Xenopus* , this gene is specifically induced in the ventrolateral mesoderm in response to bFGF [61]. Our data showed that Grb2 knockdown or miR-200a overexpression likely mediated FGF signaling that stimulated layer formation. A further suggestion was that Grb2, as a main intermediary, is indispensable to FGF signaling-mediated endoderm and mesoderm formation. miR-200a is involved in endoderm and mesoderm formation in a Grb2-dependent manner.

Previous studies have demonstrated that Grb2 transmitted FGF signaling to Erk and thus controlled the basal balance between the ES cell state and inductive differentiation [61]. Autoactivation of the Erk pathway provokes ES cell differentiation [62,63] and eliminates self-renewal [64–66]. The SH2 (Src Homology 2) domain of Grb2 fused with Son of sevenless (Sos) to induce downstream Ras activation, which specifically induces the phosphorylation and activation of Erk and rescues the endoderm differentiation phenotype in Grb2 mutant ES cells [44]. Activated Ras mutant constructs induce ES cell differentiation to the primitive endoderm layer [67], and similar results are observed in response to the expression of an activated form of Erk [68]. Erk2-null embryos results in embryonic lethality at the gastrulation and ES cell deficient in Erk1 and Erk2 is defective in mesoderm differentiation [69]. In Erk2^-/-^ ES cells, T expression is downregulated and pluripotency markers Oct4 and Nanog are maintained and they fail to differentiate into lateral mesoderm cells under mesoderm differentiation condition [62]. In neural induction in vitro, FGF-induced Erk signalling is required in a short period in the absence of BMP [62,63]. Our results showed that the extraneous addition of miR-200a significantly repressed Erk activation, due to the loss of Grb2 expression. Subsequently, endoderm and mesoderm formation was repressed, and ectoderm formation was induced. Furthermore, Grb2 supplementation rescued miR-200a-induced Erk inactivation and losses in endoderm and mesoderm differentiation after 10 days of EB formation. These findings revealed that miR-200a directly targeted Grb2, thereby mediating Erk signaling. The miR-200a-Grb2-Erk axis is therefore indispensable to layer formation in embryogenesis.

## Conclusion

Taking these findings together, we postulate that Erk signaling and miR-200a maintain a balance in specific cell fate decisions such that Erk signaling regulates differentiation into the mesoderm and endoderm lineages and miR-200a suppresses differentiation into these lineages. The link between these factors is Grb2, which delivers activation signals to Erk. Our findings suggest that miR-200a mediates Grb2 expression, thereby blocking Erk activation, which leads to the arrest of endoderm and mesoderm lineage differentiation and promotes ectoderm lineage commitment.

## Supporting Information

Figure S1 Quantifications of protein levels. (A) Quantifications of Grb2 in miR-200a and Sponge 200a treated ES cells in Figure 2C. (B) Quantifications of Grb2 in shGrb2-1 and shGrb2-2 treated ES cells in Figure 3A. (C) Quantifications of Grb2 and p-Erk in shGrb2 treated ES cells in Figure 3D. (D) Quantifications of Grb2 and p-Erk in Grb2 rescue assay in Figure 4D. All data are expressed as the means ± SD. Statistical significance was assessed by the two-tailed Student’s t test. **, p < 0.01; *, p < 0.05.
(TIF)

Table S1 Real-Time PCR primers used in this study.
(DOC)
