# Supplementary figures and images for: MicroRNA-200a Regulates Grb2 and Suppresses Differentiation of Mouse Embryonic Stem Cells into Endoderm and Mesoderm
Source: PLoS One. 2013 Jul 18;8(7):e68990. doi: 10.1371/journal.pone.0068990 (PMC3715486; doi:10.1371/journal.pone.0068990)

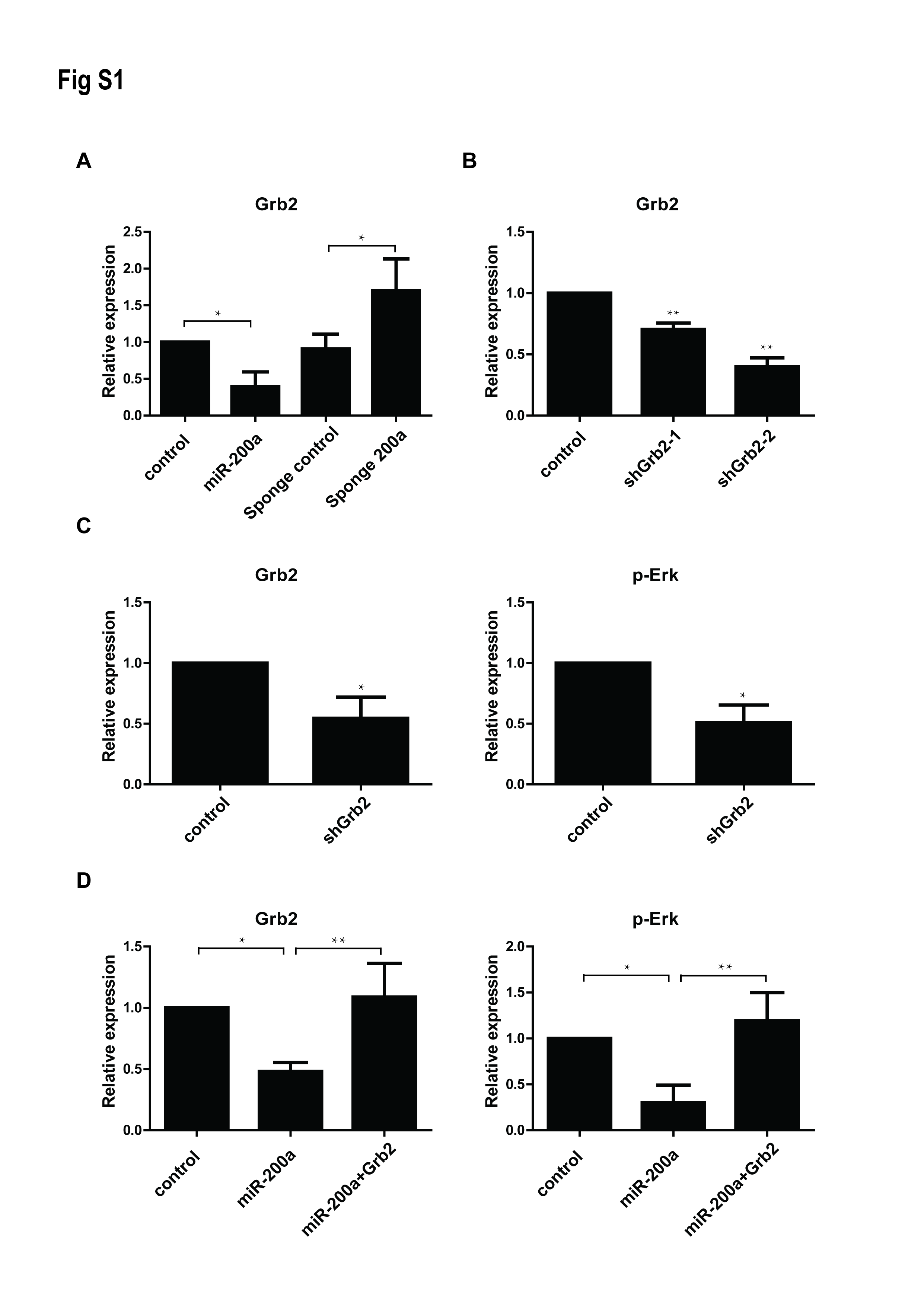

Supplement: Figure S1 — (A) Quantifications of Grb2 in miR-200a and Sponge 200a treated ES cells in Figure 2C. (B) Quantifications of Grb2 in shGrb2-1 and shGrb2-2 treated ES cells in Figure 3A. (C) Quantifications of Grb2 and p-Erk in shGrb2 treated ES cells in Figure 3D. (D) Quantifications of Grb2 and p-Erk in Grb2 rescue assay in Figure 4D. All data are expressed as the means ± SD. Statistical significance was assessed by the two-tailed Student’s t test. **, p < 0.01; *, p < 0.05. (TIF) [file pone.0068990.s001.tif]
