# Supplementary material for: MicroRNA-200a Regulates Grb2 and Suppresses Differentiation of Mouse Embryonic Stem Cells into Endoderm and Mesoderm
Source: PLoS One. 2013 Jul 18;8(7):e68990. doi: 10.1371/journal.pone.0068990 (PMC3715486; doi:10.1371/journal.pone.0068990)
Supplement: Table S1 — Real-Time PCR primers used in this study. (DOC) [file pone.0068990.s002.doc]

Table S1. Real-Time PCR primers used in this study

| Gene | Forward | Reverse |
| --- | --- | --- |
| Gapdh | GTGTTCCTACCCCCAATGTGT | ATTGTCATACCAGGAAATGAGCTT |
| Grb2 | CCCTGTCCGTCAAGTTTGGAA | GGCATCTGTTCTATGTCCCGTAA |
| Oct4 | GGATGCTGTGAGCCAAGG | GAACAAAATGATGAGTGACAGACAG |
| Nanog | CAGGTGTTTGAGGGTAGCTC | CGGTTCATCATGGTACAGTC |
| Sox2 | GATCAGCATGTACCTCCCC | CCCTCCCAATTCCCTTGTATC |
| Rex1 | GGAAGAAATGCTGAAGGTGGAGAC | AGTCCCCATCCCCTTCAATAGC |
| T | GGTGGCTTGTTCCTGGTGC | GTAGGTGGGCTGGCGTTAT |
| Alpha-Sma | ATTGTGCTGGACTCTGGAGATGGT | TGATGTCACGGACAATCTCACGCT |
| Snail | CACACGCTGCCTTGTGTCT | GGTCAGCAAAAGCACGGTT |
| Gata4 | GATGGGACGGGACACTACCTG | ACCTGCTGGCGTCTTAGATTT |
| Afp | GCCACCGAGGAGGAAGTG | AGTCTTCTTGCGTGCCAGC |
| Apoa1 | GTATGTGGATGCGGTCAAAG | TATCCCAGAAGTCCCGAGTC |
| Tuj1 | TAGACCCCAGCGGCAACTAT | GTTCCAGGTTCCAAGTCCACC |
| Pax6 | ACAGAGTTCTTCGCAACCTG | CATCTGAGCTTCATCCGAGT |
| Nestin | CTGCAGGCCACTGAAAAGTT | GACCCTGCTTCTCCTGCTC |
